# Characteristics, Treatment, and Long-Term Outcome of Gastrointestinal Involvement in Behcet's Syndrome: A Strobe-Compliant Observational Study From a Dedicated Multidisciplinary Center

**DOI:** 10.1097/MD.0000000000003348

**Published:** 2016-04-22

**Authors:** Ibrahim Hatemi, Sinem Nihal Esatoglu, Gulen Hatemi, Yusuf Erzin, Hasan Yazici, Aykut Ferhat Celik

**Affiliations:** From the Division of Gastroenterology, Department of Internal Medicine (IH, YE, AFC), Cerrahpasa Medical School, Istanbul University, Istanbul, Turkey; and Division of Rheumatology (SNE GH, HY), Department of Internal Medicine, Cerrahpasa Medical School, Istanbul University, Istanbul, Turkey.

## Abstract

Gastrointestinal involvement is rare in Behçet's syndrome (BS) patients from the Mediterranean basin. We report the demographic and disease characteristics, treatment modalities, and outcome of patients with gastrointestinal involvement in BS (GIBS).

We retrospectively reviewed the charts of all BS patients in our BS clinic with a diagnosis of GIBS. Patients were invited to the clinic to assess their outcome.

Among 8763 BS patients, we identified 60 with GIBS (M/F: 32/28, mean age at diagnosis: 34 ± 10, mean follow-up: 7.5 ± 4 years), after excluding 22 patients with mimicking symptoms. Six (10%) had juvenile-onset BS. The most common intestinal localization was ileocecal region (36/59, 61%) mainly as big oval ulcer/s. Initial treatment was azathioprine for moderate to severe (n = 37) and 5-ASA for mild cases (n = 16). Anti-TNFs and/or thalidomide provided remission in 12 of 18 (67%) refractory patients. Emergency surgery was required in 22 patients. Nine patients did not receive postoperative immunomodulators and 8 relapsed. Overall, 48 of 60 (80%) patients were in remission (29/48 without treatment) at the time of survey. Three recently treated and 2 refractory patients were still active, 3 had died due to non-GI-related reasons, and 4 were lost to follow-up.

Careful evaluation for excluding mimickers is important during the diagnosis of GIBS. Azathioprine seems to be a good choice as first-line treatment with high remission rates and few adverse events. Thalidomide and/or TNF-alpha antagonists may be preferred in resistant cases. Surgery may be required for perforations or massive bleeding, and postoperative immunosuppressive treatment is necessary for preventing postoperative recurrences.

## INTRODUCTION

Behçet's syndrome is a multisystem vasculitis that can involve the skin, mucosa, joints, eyes, veins and arteries, central nervous system, and the gastrointestinal (GI) system.^1^ GI involvement can be a life-threatening complication in BS due to perforations and massive bleeding.^2^

The frequency of GI involvement in BS shows important variations across geographies. It is reported as high as 30% among Japanese and Korean BS patients, compared to 1% to 2% in countries around the Mediterranean basin including Turkey and between 5% and 20% in European countries.^2^ Moreover, we had a reason to suspect that there could be geographical differences in disease expression, as well, because such differences were observed in reports from different countries.^3^ As far as we know, the only reports on characteristics of gastrointestinal involvement came from the Far East. We were unaware of the previous case series other than those from the Far East, reporting demographic features, treatment modalities, treatment response, natural course, and prognosis of patients with gastrointestinal involvement in Behçet's syndrome (GIBS). Such information is important in disease management because current management strategies in patients from geographies with low frequency of GIBS are tailored by extrapolating data from the available evidence for other inflammatory bowel diseases, mainly Crohn's disease (CD).^4^

We thus surveyed the demographic and disease characteristics, treatment modalities, and long-term outcome of our BS patients with GI involvement attending our multidisciplinary, dedicated clinic.

## METHODS

We performed a retrospective chart review of all of our GIBS patients. We used a standard form to recover the demographic features, other BS manifestations, clinical findings, endoscopic findings, histologic findings, treatment modalities, and outcome.

BS patients who develop GI symptoms are evaluated by the gastroenterologists in our multidisciplinary BS clinic and also who run the inflammatory bowel disease clinic where CD, ulcerative colitis (UC), GIBS, and gastrointestinal tuberculosis patients have been recorded in the same database for >15 years. All BS patients with possible GI involvement undergo endoscopic examination and those with ulcers are further scrutinized to exclude other causes such as nonsteroid anti-inflammatory drugs (NSAIDs) use. NSAIDs are stopped and if the symptoms disappear, endoscopy is repeated within 2 to 3 months to confirm that the ulcers have healed. Patients with BS and inflammatory GI involvement are defined as GIBS, including those with morphological resemblance to either CD or UC.

**FIGURE 1:**
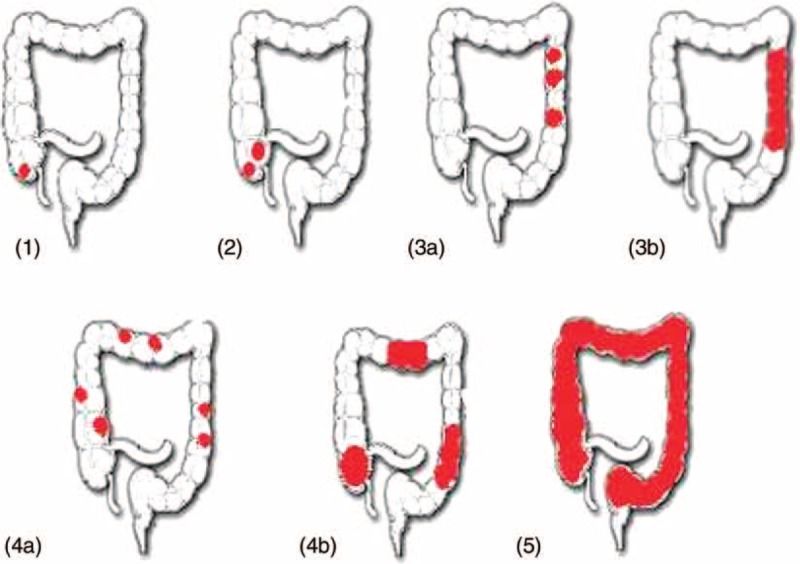
The types and the frequency of the extent of endoscopic ulcers. Footnotes: 1) Focal single; 2) Focal multiple; 3) Segmental, (3a) multiple and (3b) diffuse; 4) Multisegmental (4a) Multiple and (4b) diffuse; 5) Pancolitis.

**FIGURE 2:**
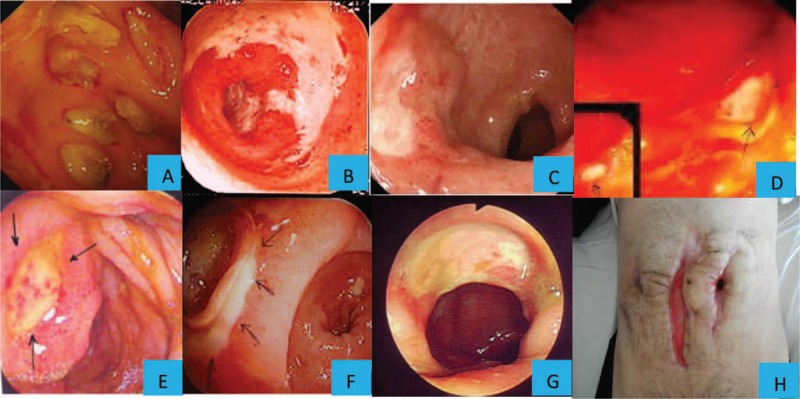
The types of endoscopic ulcers in gastrointestinal Behçet's syndrome. (A) Multiple oval/round ulcers; (B) Geographic type ulcer; (C) Oval shaped ulcer; (D) Aphtous ulcer; (E) Oval shaped ulcer with elevated edges and hematin spots on the center; (F) Oval shaped ulcer at anostomosis; (G) Oval shaped large ulcer (H) Recurrent perforations following right hemicolectomy, possibly due to a pathergy phenomenon following surgical trauma.

The extent of ulcers was determined by a combination of available endoscopic and surgical records and categorized as follows: focal single, focal multiple, segmental, multisegmental, and pancolitis (Figure 1). In addition, we divided segmental and multisegmental involvement into 2, according to the type of the ulcers as diffuse and multiple. We also recorded the shape of ulcers as round/oval, geographic, star-shaped, aphthous, linear, or ulcerative colitis-like (Table 2, Figure 2A–G). We also reviewed the histopathologic findings of endoscopic and surgical excision biopsies, and noted the presence of inflammation, granuloma, vasculitis, crypt distortion, and thrombus formation.

**TABLE 2:**
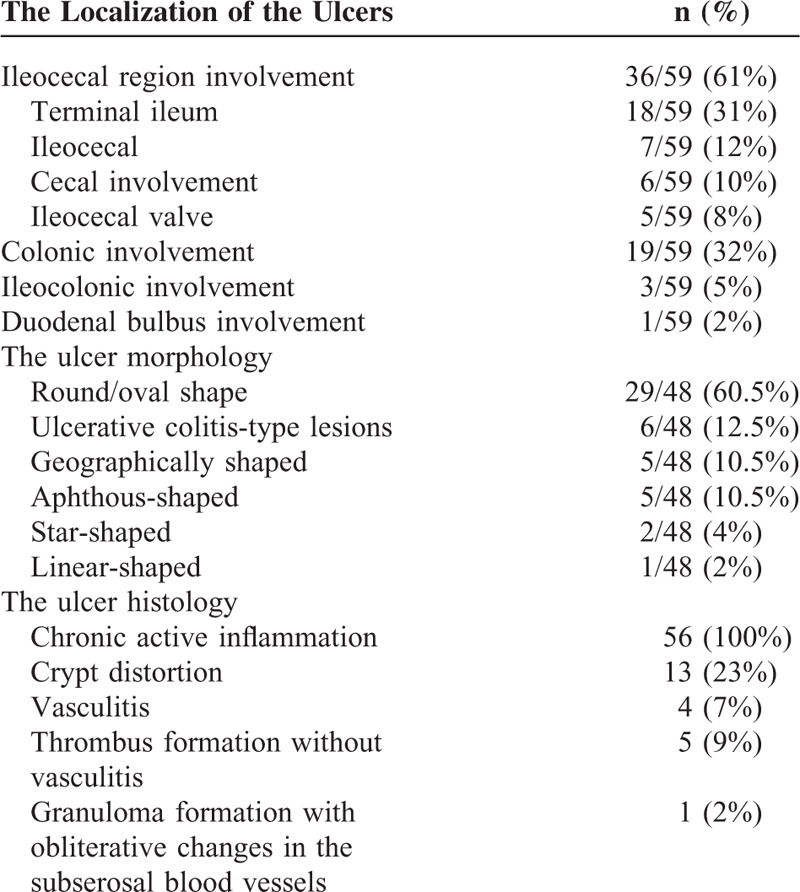
The Localization, Morphology, and Histologic Findings of the Ulcers

The doses and duration of drugs used and the responses were recorded. Surgical procedures were confirmed from the records of the surgery department. The outcome of patients who were not under regular follow-up was assessed by calling the patients to our outpatient clinic for a final evaluation. If the patient lived in another city and was not able to come, they were questioned by phone calls, and their physicians were contacted if necessary. We classified a patient as in remission if the patient had no GI symptoms and had no GI ulcers on the final control endoscopy. If the patient had no symptoms and did not consent to having a control colonoscopy, we determined the calprotectin levels in stool and C-reactive protein (CRP) levels in blood to exclude the presence of inflammation. All relapses were endoscopically confirmed. The Ethics Committee of Cerrahpasa Medical Faculty approved the study.

## RESULTS

**FIGURE 3:**
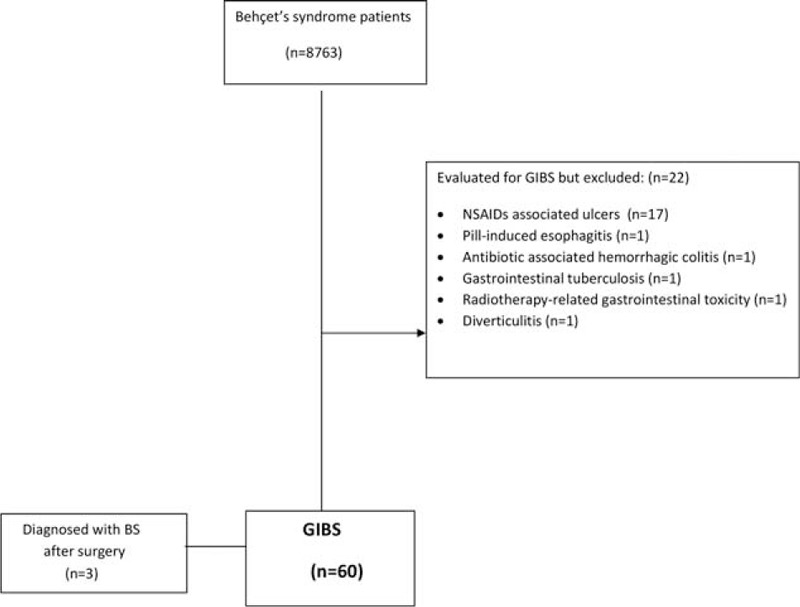
Study flow diagram.

Among the 8763 BS patients treated in our multidisciplinary BS clinic at Cerrahpasa Medical Faculty, we identified 60 GIBS patients who were studied between 1980 and March 2015. Three of these patients did not have a previous diagnosis of BS and were diagnosed as BS only after gastrointestinal involvement developed. Nineteen of these 60 patients were diagnosed as GIBS after emergency surgery for perforations or massive bleeding (Figure 3).

In addition to these 60 GIBS patients, 22 BS patients had been evaluated with a suspicion of gastrointestinal involvement and were found to have other reasons for their gastrointestinal symptoms and endoscopic lesions. These reasons were NSAID ulcers in 17 patients, pill-induced esophagitis, antibiotic-associated hemorrhagic colitis, gastrointestinal tuberculosis, radiotherapy-related gastrointestinal toxicity, and diverticulitis in each 1 patient (Figure 3).

The demographic and clinical features of patients are given in Table 1. Six (10%) BS patients were <16 years at disease onset. Three (5%) patients had sacroiliac inflammation on magnetic resonance imaging (MRI), which was not detectable with x-ray in 2 of 3 patients.

**TABLE 1:**
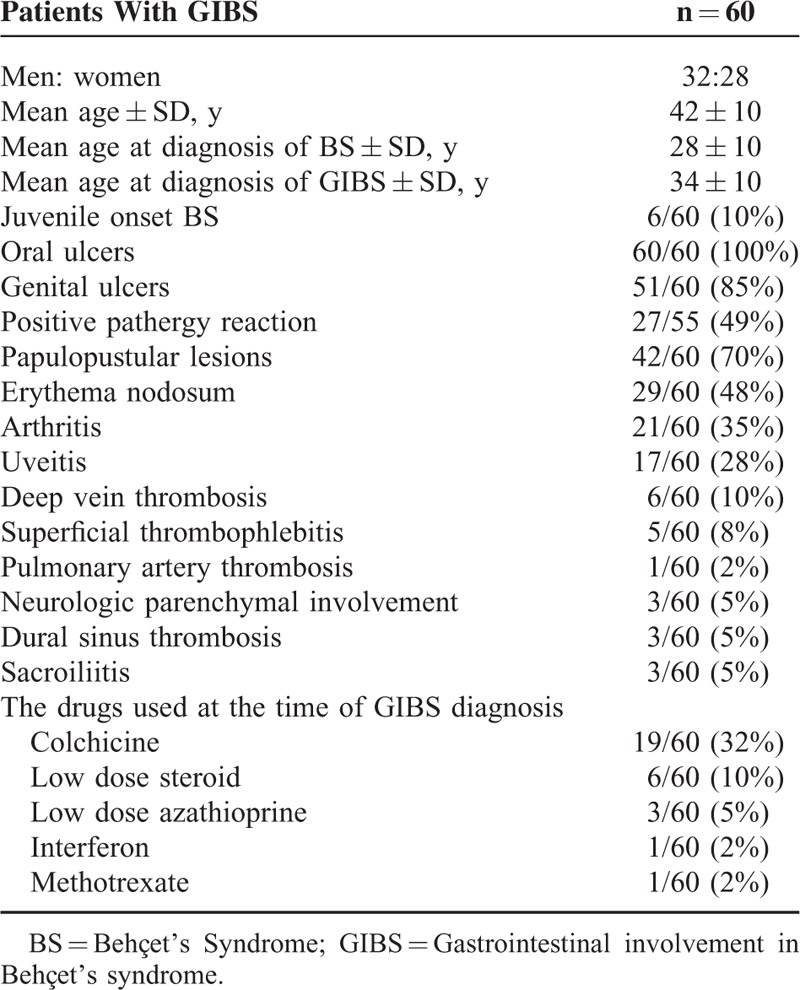
Demographic Features and Other Manifestations of Participants

### Clinical Features

The most common presenting symptoms were abdominal pain (52/60, 87%) and diarrhea (29/60, 48%). Nineteen (32%) patients presented with an acute abdomen. Eight of these patients (42%) had massive bleeding and 11 (58%) had perforations at presentation. One additional patient with initial massive bleeding developed a perforation during colonoscopy. Among the 11 patients with perforations, 8 were free and 3 were closed perforations.

Five patients (8%) had enterocutaneous fistula postoperatively. Three (5%) had simple perianal fistulae and 1 (2%) had an enteroenteric fistula. Among the patients who were on medical treatment for GIBS, 3 experienced perforations and 1 had an enterocutaneous fistula.

Data regarding fever were available for 51 GIBS patients. Thirty-eight (74.5%) of these had no fever at the onset of GIBS, whereas in the remaining 13 (25.5%) fever was one of the initial symptoms. Among these 13 patients, 11 had acute abdomen during the fever. The other 2 patients with fever had concomitant myelodysplastic syndrome associated with trisomy 8. Among the 12 patients who experienced relapses, 3 (25%) had fever at the time of their relapse.

### Acute Phase Response

Data regarding acute phase reactants during presentation were available for 50 GIBS patients. CRP level during presentation was high in 39 (78%) of these patients. The mean ± SD CRP level in patients who had to undergo surgery was significantly higher than those who did not (157 ± 85 vs 33 ± 19; *P* < 0.001).

### Endoscopic and Surgical Findings

The typical endoscopic appearance was the presence of ulcers of variable shape and type on a relatively normal mucosal background. Round/oval-shaped ulcers were the most frequent lesions, observed in 29 of 48 (60.4%) patients. Among these 29 patients, 13 (45%) had a single large (>1 cm) ulcer. In 2 of the patients with round/oval-shaped ulcers, few aphthous and linear-type ulcers were also observed. The most common type of involvement was a focal single ulceration, observed in 21 of 57 (37%) of the patients (Figure 1). The most frequent localization of involvement was the ileocecal region (36/59, 61%) (Table 2).

### Histologic Findings

Endoscopic biopsies from 36 patients and surgical excision biopsies from 20 patients were available (Table 2). All specimens showed chronic active inflammation. Crypt distortion was found in 13 (23%) patients. Vasculitis was present in 4 (7%) of the biopsies (3 surgical specimens and 1 endoscopic specimen). Thrombus formation without vasculitis was present in 5 (9%) of the surgical biopsies. Granuloma formation with obliterative changes in the intestinal subserosal blood vessels was observed in 1 (2%) patient.

### Treatment Modalities

Among the 60 patients with GIBS, 19 (32%) had emergency surgery due to perforation (n = 11) or severe lower GI bleeding (n = 8), which were mutually exclusive, before any treatment was started. In addition, 3 patients underwent emergency surgery while they were on medical treatment. Ten of the 19 patients who required emergency surgery were treated with immunosuppressives after the operation. The remaining 9 patients were not given medical treatment after surgery and 8 of 9 experienced relapses. The relapse was managed with medical treatment in 7 of the patients and with reoperation without postoperative medical treatment in 1 patient.

The initial medication was azathioprine 2 to 2.5 mg/kg/day in 37 patients, 5-ASA compounds in 16, infliximab in 3, and budesonide in 2 patients. Two patients who had to undergo emergency surgery did not receive medical therapy postoperatively. In addition to these, prednisolone 0.5 to 1 mg/kg/day was used in 10 patients during acute exacerbations with severe symptoms and 4 patients used short-term prednisolone for other BS manifestations such as eye involvement.

#### Azathioprine

A total of 44 patients were prescribed azathioprine either as first line (n = 37), or after nonresponsiveness to 5-aminosalicyclic acid (5-ASA) or budesonide (n = 7). Among the 37 patients who were prescribed azathioprine initially, remission was observed and there were no relapses in 24 (65%) patients during a mean follow-up of 68.6 ± 43.6 months. Eleven of these had undergone emergency surgery before starting azathioprine. One patient received additional cyclosporine-A owing to uveitis and the other one was treated with additional 5-ASA compounds. Eight patients were refractory to azathioprine. Among these 8 patients, 4 had remission with thalidomide and 1 with infliximab. One patient was refractory to thalidomide, infliximab, and adalimumab and was diagnosed to have myelodysplastic syndrome involving trisomy 8 and obtained remission after allogeneic bone marrow transplantation.^5^ One patient who was refractory to azathioprine underwent 6 surgical operations for postoperative complications and died with complications of end-stage renal disease due to amyloidosis. The other patient who was refractory to azathioprine and infliximab additionally developed a pulmonary artery thrombosis and died. Three patients had to have ileal resections due to perforation of the ileum. In 2 of them, azathioprine was continued postoperatively and they are still in remission with azathioprine. The other was left untreated after surgery and had a relapse 6 years after the operation. Azathioprine was started, but remission could only be achieved with infliximab. Finally, 1 patient was lost to follow-up and the other started azathioprine recently.

**FIGURE 4:**
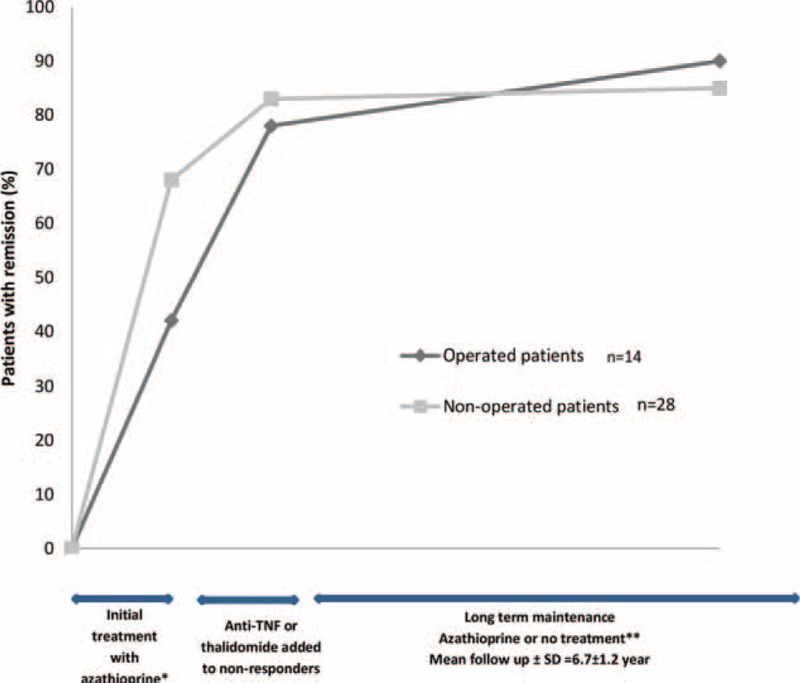
Remission rates over time in patients who were started azathioprine. ^∗^Five of these patients were treated with 5-ASA or budesonide before azathioprine and were resistant to these. Two additional patients who had recently started azathioprine were excluded for the analysis. ^∗∗^Two patients were lost to follow-up after remission and 1 had remission with allogeneic bone marrow transplantation.

We also tabulated the patients who had remission under azathioprine according to having emergency operation or not, after excluding the 2 patients who had started azathioprine very recently (Figure 4).

#### 5-ASA Compounds

Among the 16 patients who were given 5-ASA compounds, 10 (62.5%) achieved remission and did not relapse during the 89.3 ± 64.5 months that they were followed. One patient who initially had remission with 5-ASA relapsed after 3 years, developed myelodysplastic syndrome involving trisomy 8, and died due to invasive pulmonary aspergillosis during high corticosteroid therapy for GI relapse. Azathioprine was started in 5 patients who were refractory to 5-ASA compounds, together with infliximab in 2 of them. Two patients who were prescribed infliximab and azathioprine and 1 of the patients on solo azathioprine obtained remission with these agents. The other patient was refractory to azathioprine and was switched to infliximab that also did not provide remission. Adalimumab was started and remission was obtained. Adalimumab was stopped 11 months later and she is still doing well with only azathioprine. The fifth patient was switched from azathioprine to adalimumab owing to azathioprine toxicity and is currently continuing adalimumab.

#### Infliximab

Infliximab was the initial choice of treatment in 3 patients with severe lesions. One of them was prescribed concomitant azathioprine and achieved remission with this treatment, the other started infliximab only recently and is doing well, and the final patient was refractory to infliximab monotherapy and obtained remission when thalidomide was added.

#### Budesonide

Initial treatment was with budesonide only in 2 patients who had ileal involvement. One was refractory to budesonide and additional azathioprine, and remission could only be obtained when adalimumab was added. The other one received only 3 months of this treatment and was lost to follow-up. He came back to our clinic without GI symptoms 4 years later. Control colonoscopy revealed a huge (4 cm) ileal ulcer and azathioprine was started.

### Surgical Treatment

The 19 surgical procedures that were performed in patients who did not have previous medical treatment were right hemicolectomy in 10 patients, ileocecal resection in 3, total colectomy in 2, left hemicolectomy in 2, ileal resection in 1, and subtotal stomach resection in 1 patient. Three additional patients (5%) had to go undergo surgery due to perforations despite treatment with azathioprine for 6 months, 1 year, and 7 years after the initiation of medical treatment. Thus a total of 22 patients required surgery. In 13 patients, immunosuppressives (azathioprine in 10 patients, 5-ASA compounds in 2 patients, and combination of infliximab and azathioprine in 1 patient) were initiated within 1 to 2 months following surgery depending on wound healing. One of these patients received corticosteroids in addition to azathioprine. The remaining 9 patients did not receive medical therapy after surgery.

Five patients had to have a reoperation. Two of these patients had had right hemicolectomy initially, and were later operated for enterocutaneous fistulas while being on azathioprine therapy, 3 and 4 months after the initial operation. One of them continued to receive azathioprine therapy and the other was treated with infliximab in addition to azathioprine following reoperation. One patient had undergone a truncal vagotomy and gastroenterostomy and 6 months later being off treatment had to have a subtotal gastrectomy with roux-en-y reconstruction due to a large ulcer at the anastomosis site, and azathioprine was initiated but remission was obtained only after the addition of thalidomide therapy. One patient who had had a right hemicolectomy needed a reoperation for stenosis due to a giant ulcer at the anastomosis site 4 years later. This patient was on azathioprine therapy at the time of reoperation and azathioprine was continued after reoperation. One patient who was on azathioprine needed 6 operations following a right hemicolectomy due to recurrent perforations, possibly due to a pathergy phenomenon following surgical trauma (Figure 2H). Thus, all of the patients who required reoperation had been given immunosuppressives after the initial surgery and except for 1 patient, all of them were still using immunosuppressives when reoperation was required.

### Outcome

At the end of a mean follow-up of 7.5 ± 4 years (range 0.1–19.2 years), 3 (5%) patients had died owing to non-GI-related reasons, 5 (8%) patients were still active (3 of them were newly treated patients), 48 (80%) patients were in remission, and 4 (7%) patients were lost to follow-up.

The relapse rate during our follow-up was 12 of 60 (20%). Among the 48 patients who were in remission at the time of this survey, 29 of them (60%) were off treatment. Among the 48 patients who were in remission, this was confirmed by colonoscopy in 45. Absence of clinical findings together with low fecal calprotectin levels suggested remission in the remaining 3 patients. Regarding the number of patients requiring surgery and the number of patients requiring treatment with thalidomide or TNF-alpha antagonists indicating more severe GIBS, there was no difference between men and women (*P* = 0.47 and *P* = 0.07, respectively) and between patients with a young age at onset (<25) and older onset (*P* = 0.51 and *P* = 0.42, respectively).

The reasons for death were pulmonary artery thrombosis, infection, and acute renal failure due to amyloidosis in 1 patient each. Among the 4 (7%) patients who were lost to follow-up, 1 was still active and 3 were in remission during their last visits to our clinic. Among the 5 active patients, 3 were newly registered patients. Myelodysplastic syndrome (MDS) (n = 2) and polycythemia vera (n = 1) were observed in 3 patients. All of these 3 patients with hematologic disorders also had trisomy 8.

## DISCUSSION

Gastrointestinal involvement of Behçet disease is less common in Turkey than in the Far East and the United states.^2^ Although this study was not a formal analysis of the prevalence of gastrointestinal involvement in BS, among our 8763 patients with BS, there were only 60 GIBS patients (0.7%). This is much lower than that observed in Japan and Korea.^2^ In addition to the possibility of a real difference in the frequency of GIBS across geographies, there are other possible reasons for this difference such as differences in diagnostic methods such as endoscopy, radiology, or only clinical findings; differences in specialties such as reports from gastroenterology, rheumatology, dermatology, or ophthalmology departments; increased awareness in the Far East resulting in increased referral; inclusion of GI involvement in the Japanese criteria for BS in contrast to the International Study Group for Behçet Disease criteria; misdiagnosis of drug-induced ulcers (with agents such as NSAIDs or possibly colchicine) as GIBS.^2^ Our report especially underlines the importance of excluding other reasons that may mimic GIBS, because 22 BS patients who had GI symptoms and ulcers on endoscopy were diagnosed to have other causes for these.

The most striking demographic finding of our study was the high frequency of juvenile onset patients (10%). The frequency of childhood onset was 0.8% in our general BS population.^6^ Our juvenile onset GIBS patients tended to have a more severe course, 3 of 6 required TNF-alpha antagonists and 1 required thalidomide, resulting in remission in all. As most of our patients were in remission at the end of the follow-up period, we could not report a probable sex or juvenile/adult onset difference on the influence of long-term prognosis of GIBS. Interestingly, GIBS is not very common and does not seem to run a more severe course among the male patients. This is different from the other types of major organ involvement in BS. There are conflicting observations regarding male and female predominance in previous reports on GIBS from the Far East.^7–10^ However, a meta-analysis of 52 datasets found no gender difference for GIBS, in contrast to other manifestations.^11^ Regarding other BS manifestations, we observed that the frequency of ocular involvement was less among our GIBS patients than the frequency in our general BS population (28% vs 47%).^12^ A similar observation was reported by our Japanese and Chinese colleagues.^13,14^ Interestingly, 3 (5%) of our patients had sacroiliitis on MRI, a feature of inflammatory bowel disease rather than BS. One patient had HLA B27. However, we do not know the HLA B27 status of the other 2 patients.

Our survey showed that presentation with perforations and gross rectal bleeding are important features of GIBS (18% and 13%). It is generally thought that gastrointestinal complications such as perforations and massive bleeding are common in GIBS due to the presence of large and deep ulcers.^15,16^ Perforations or massive bleeding in our survey was more common than what we had previously observed in our patients with CD, occurring in almost one third of the patients with GIBS and 2.5% with CD.^17^ This may be indicative of a vasculitic process in GIBS. However, we were able to show vasculitis in only 7% of the biopsies in our patients. Perforations, bleeding, round/oval-shaped ulcers, and focal distribution in GIBS should raise the suspicion of GIBS rather than CD.^10,18^ Especially in low-prevalent countries for BS, the differences can be easily overlooked by gastroenterologists. Moreover, CD shares other manifestations with BS such as oral ulcers, arthritis, and erythema nodosum.^19^ Thus, a detailed consideration of BS is important in all GIBS patients, in order not to miss the diagnosis of BS and leave other BS manifestations untreated.

Our general approach in the management of GIBS patients is to start with 5-ASA derivatives in patients with mild superficial ulcers and with azathioprine in patients with moderate to severe clinical and endoscopic findings. Prednisolone may be added for short durations during severe acute exacerbations. In resistant cases, thalidomide and/or TNF-alpha antagonists are used. The TNF-alpha antagonists that we use are infliximab and adalimumab that may be interchanged when there is lack of efficacy or adverse events. With this strategy, the prognosis of our GIBS patients was generally good. Only 5 patients were still active and 3 of these were new patients within the first 3 months of treatment. Among the 2 patients who died, 1 had died owing to another BS-related manifestation, pulmonary artery thrombosis. Three of the 4 patients who were lost to follow-up were in remission during their last visit to our clinic.

This treatment approach is somewhat different from the recently published revised recommendations for the management of GIBS by our Japanese colleagues.^20^ They recommended 5-ASA compounds for induction therapy of patients with mild to moderate symptoms and corticosteroid and/or TNF-alpha antagonists for induction therapy of patients with severe symptoms and 5-ASA compounds and colchicine for maintenance therapy. Azathioprine was reserved for corticosteroid-dependent or -resistant patients. Differing from our Japanese colleagues, we think azathioprine is a good choice as first line therapy because 65% of our patients treated with azathioprine obtained remission and did not relapse during a mean follow-up of nearly 6 years. Moreover, it seems to be safe in this group, because there was only 1 patient who had to discontinue azathioprine owing to an adverse event, a transaminase elevation. In addition, our relapse rate was 20%, somewhat lower than that reported from the Far East countries.^21^ The second difference is in the use of prednisolone. The Japanese recommendations suggest that prednisolone may be used solo in severe patients. We always use corticosteroids in addition to immunosuppressives and try to taper it as quickly as possible, similar to what is usually done in other inflammatory bowel diseases. There has been a concern about prolonged healing of ulcers, bowel perforation, and perioperative complications due to corticosteroid use in GIBS patients.^22^ It was previously observed that corticosteroid use was associated with higher operation rates in an univariate analysis, but this was not confirmed in a multivariate analysis.^23,24^ We used corticosteroids in addition to immunosuppressives in 14 patients with severe acute exacerbations and did not observe any perforations. The third difference is in TNF-alpha antagonists that we reserve for patients who are refractory to standard therapy in contrast to our Japanese colleagues who use it as first line therapy. Our main concern regarding the use of TNF-alpha antagonists is a high tuberculosis rate reported in BS patients.^25^ Moreover, we think thalidomide may be an alternative to TNF-alpha antagonists, because among the 6 azathioprine-resistant patients and 1 infliximab-resistant patient who were prescribed thalidomide, only 1 did not obtain remission and had to switch to infliximab. Finally, colchicine is not a drug that we prefer in major organ involvement of BS including GIBS. Moreover, it has a well-known potential to cause diarrhea and also has been considered to be related to GI ulceration.^22,26^

Emergency surgery was required in 22 (37%) of our GIBS patients, and in 19 of them surgery was required at presentation. Almost half of the patients who were operated experienced relapses. Other groups also reported a high relapse risk after surgery.^27^ This high relapse risk may be related to the pathergy phenomenon that was also reported to cause postoperative complications after vascular surgery, such as aneurysms at anastomosis sites.^27,28^ Concomitant immunosuppressive use seems to be necessary in these patients to prevent postoperative recurrences. We observed that among the 9 patients who did not receive medical therapy after surgery, 8 (89%) had relapses. This finding supports using maintenance therapy after surgery to reduce the risk of recurrence in GIBS patients.

The association between MDS, trisomy 8, and BS, especially with gastrointestinal involvement, has been well recognized.^29^ These are generally more severe patients, resistant to immunosuppressives, as we and others have observed.^30^ Whether the presence of trisomy 8 with or without MDS plays a role in the pathogenesis of GIBS or contributes to the refractoriness of GIBS to medical treatment is not clear.

Our survey had some limitations. This was a retrospective study and although all cases had been consulted by the senior gastroenterologist who has been running the clinic from the beginning, endoscopies were not performed by the same doctor and not formally evaluated to determine the specific ulcer types and localizations. The endoscopic pictures, endoscopy reports, and/or macroscopic descriptions of surgical specimens were scrutinized by 2 of the authors together (AFC and IH) to determine the ulcer types. Second, we had to rely on stool calprotectin levels for determining remission in 3 patients who were asymptomatic and did not consent to having a control endoscopy. However, there is at least 1 meta-analysis indicating that fecal calprotectin levels are useful for the assessment of bowel inflammation.^31^ In addition, we determined the localization of involvement by endoscopy which can visualize only a small part of the small intestine. Capsule endoscopy or double balloon enteroscopy may be more reliable methods; however, they may cause an overestimation of gastrointestinal involvement by detecting clinically irrelevant lesions that may cause unnecessary treatment. Finally, the histopathologic assessment of all colon biopsies and resection materials were not evaluated by the same pathologist.

In conclusion, in this first GIBS series outside the Far East, we stressed on a high frequency of presentation with perforation and massive bleeding, a high frequency of focal distribution and oval-shaped ulcers in the ileocecal region, and a severe disease course in juvenile onset patients. We observed that conditions mimicking GIBS such as NSAID ulcers are common among BS patients and must be excluded before diagnosing GIBS. Postoperative immunosuppressive treatment seems to be vital for decreasing postoperative recurrences. Azathioprine seems to be a good choice as first-line with high remission rates and few adverse events. Thalidomide and/or TNF-alpha antagonists may be preferred in resistant cases. The optimal mode and duration of maintenance therapy, once remission is obtained is not clear. Clinical and endoscopic findings are similar with the other reports from Far East, whereas our relapse rate are lower and treatment strategies are different with the use of azathioprine as a first-line treatment and with avoiding corticosteroids and colchicine.
